# Delay Analysis in IoT Sensor Networks †

**DOI:** 10.3390/s21113876

**Published:** 2021-06-04

**Authors:** Asaad Althoubi, Reem Alshahrani, Hassan Peyravi

**Affiliations:** 1 Department of Computer Science, Kent State University, Kent, OH 44242, USA; aalthoub@kent.edu; 2 Department of Computer Science, Taif University, Taif 26571, Saudi Arabia; rashahrani@tu.edu.sa

**Keywords:** sensor networks, edge networks, data center networks

## Abstract

Internet of Things (IoT) devices, particularly those used for sensor networks, are often latency-sensitive devices. The topology of the sensor network largely depends on the overall system application. Various configurations include linear, star, hierarchical and mesh in 2D or 3D deployments. Other applications include underwater communication with high attenuation of radio waves, disaster relief networks, rural networking, environmental monitoring networks, and vehicular networks. These networks all share the same characteristics, including link latency, latency variation (jitter), and tail latency. Achieving a predictable performance is critical for many interactive and latency-sensitive applications. In this paper, a two-stage tandem queuing model is developed to estimate the average end-to-end latency and predict the latency variation in closed forms. This model also provides a feedback mechanism to investigate other major performance metrics, such as utilization, and the optimal number of computing units needed in a single cluster. The model is applied for two classes of networks, namely, Edge Sensor Networks (ESNs) and Data Center Networks (DCNs). While the proposed model is theoretically derived from a queuing-based model, the simulation results of various network topologies and under different traffic conditions prove the accuracy of our model.

## 1. Introduction

The exponential growth in wireless and mobile devices has resulted in producing an unprecedented amount of traffic that needs to be processed by various nodes, sometimes in real time. The advancements in sensing, data processing, and cloud communication technology have enabled the systems to interact with the environment and optimize processes. The Internet of Things (IoT) is evolving from vertical to polymorphic applications, supporting both personal and industry users. To provide a seamless experience to the end-users, many challenges need to be addressed, including technology standards, interoperable module components supporting heterogeneous applications and requirements at several layers. In general, scalability is a limiting factor both vertically and horizontally. In the vertical scalability, computing resources are added or deleted, and in the horizontal scalability, network nodes are added or deleted. Security, privacy, self-organization, and energy efficiency are also other challenges in IoT deployment. The vehicular networks applications, such as Vehicular to Vehicular (V2V), Vehicular to Pedestrian (V2P), and Vehicular to Infrastructure (V2I) networks are latency-sensitive networks. Some healthcare and wearable devices have stringent requirements in terms of latency and latency variation (jitter). The authors in [1] provide a survey of communication protocols for IoT networks and related challenges. In [2], a taxonomy of IoT broad vision for various applications is examined. It provides a categorization of analytic approaches and proposes a layered taxonomy from IoT data for analytics.

Several billion active IoT and sensor devices around the world continuously require data connections to data centers to operate and connect with other devices. These connections to data centers yield a significant ever-growing amount of data continuously being updated as the use of IoT increases. How is this affecting data centers around the world? Data storage is a very important consideration. With such a huge rise in data consumption and traffic, the connections from devices to data centers will need to be improved drastically in terms of response time, particularly for real-time applications. Real-time applications include environmental sensor networks, weather forecasting networks, healthcare, and epidemic networks, etc. With more smart houses being built than ever before and more IoT devices being implemented, data centers will need to respond within a constrained time.

These applications service several millions of queries and instructions on several thousands of machines and are concerned with *response latency*, *latency variation* (jitter), and *tail latency*. Optimizing data center networks for tail latency is a shift from the previous designs where the performance metrics of interest were throughput or average latency. Moreover, optimizing for tail latency is another key performance metric and has already been considered in vertical designs with new operating systems, cluster managers, and data services [3,4,5]. Optimizing the average throughput for latency-insensitive traffic is still an important performance metric.

Network latency is directly related to the two other troublesome network complications, packet loss and jitter. Thus, identifying the root causes of the latency problem and what latency means to the applications is necessary. First, request latency reflects how long it takes from sending a request to receiving the reply, including the time spent waiting in the queue and the time spent executing the corresponding instructions. Second, a significant portion of latency is dominated by the network transfer time which in turn is dominated by the queuing probabilistic delay. There is also the variation in the network latency, which is caused by various network traffic types, including the background traffic and the request/response traffic with tight deadlines. The background traffic can be latency-sensitive packets, short control messages, or large replicated transfer files. Third, the absence of priority scheduling for latency-sensitive traffic when latency-sensitive and non-latency-sensitive traffic share the network equally. Additionally, uneven load balancing and burst traffic all contribute to variation in network transfer times. Fourth, increasing buffer size to reduce loss rate introduces variable buffer latency. As predictability of response time becomes more critical for latency-sensitive cloud services, finding a balance between the expected response time and deployment of computation/communication resources becomes more critical.

In this paper, an analytical model was developed to reduce the stringent task service delays for sensor and IoT devices, particularly when they must obtain or update data from/to cloud data centers. Eliminating the sources of latency and latency variability in large-scale systems is impractical, especially in shared environments. However, predictions and mitigation of latencies are possible through queuing analysis.

Our main contributions can be summarized as follows:

- We developed a two-stage tandem queuing model for Edge Sensor Networks (ESNs) and Data Center Networks (DCNs), as well as feedback mechanisms to investigate key performance indicators, such as latency, delay variation (jitter), utilization, and the optimal number of computing units needed in a cluster.
- We derived closed-form formulas for the above key performance indicators and analyzed tail latency under various traffic scenarios. We extended the result presented in [6] for the last mile delay analysis in DCN networks to incorporate the first-mile delay analysis for ESNs and achieve end-to-end delay analysis.
- We formulated the compound latency accumulated by ESNs and DCNs, which can be used to suggest the optimal workload allocations among schedulers and servers, thereby minimizing the turn-around time required by applications.
- These findings were verified by simulating the system with the traffic obtained from synthetic data as well as IoT traffic traces. The distinction between the model presented in this paper and the previous models [7,8,9,10] is three-fold. First, the model relaxes the assumption in some previous models in which the servers were assumed to be homogeneous to be able to apply an M/M/k queuing model. However, in our model, the servers are assumed to be heterogeneous with different service rates. Second, unlike some previous models [7,8] and from the queuing perspective, the model separates schedulers from servers. Distributed schedulers perform distinctive operations and could cause a bottleneck. With heterogeneous servers, different servers have different probabilities of being dispatched depending on their load. Third, since scheduling policies are based on fair sharing through statistical multiplexing and/or over-provisioning, the previous models were not capable of providing guarantees on job latency.

### Source of Latency and Challenges

Generally, the network-specific real-time analytics, heterogeneous resources, scalability of many detailed data, tail latency, and sub-millisecond response time requirements represent most of the critical challenges facing data centers. Short-running jobs are normally held disproportionately and become subordinate to long-running jobs (called stragglers [11]). Despite using many straggler mitigation techniques, stragglers are still increasing the average job duration by 47%, which means the job completion suffers significantly from stragglers [11]. A connection is only useful if it satisfies its deadline. However, stragglers increase tail latency, which can also be influenced by resource contention with background jobs, device failure, uneven split of data between tasks, and network congestion.

A query or response initiated by a sensor or an IoT node suffers a cascade of three latencies. The edge latency is associated with an edge distribution network consisting of edge devices such as computers, Wi-Fi access points, and desktop and wiring closet switches, also known as hosts or end systems, which are connected at the edge of the network. The core network latency is associated with operation in data centers. Data centers have their edge networks in racks that aggregate server traffic. Finally, network latency is often dominated by propagation (distance) delay between the edge network and core network (data centers). The first mile (edge network) and the last mile (data center or core network) contribute significantly to the latency and its variation. In the following sections, we develop models for edge and core networks to estimate and predict the latency and latency variation in each network. The edge network model consisting of sensors and IoT devices is called the Edge Sensor Network (ESN), and the core network model associated with a cloud center is called the Data Center Network (DCN).

An analytical model is a valuable tool that provides insights into a system’s behavior under normal and extreme conditions. One can draw a trajectory of the system’s characteristics by varying the system’s parameters with extreme or boundary values. For example, one can increase the load or aggregation level to project tail latency. This is particularly useful when schedulers target real-time data processing in which jobs are latency sensitive. The Data Center Network (DCN) is modeled based on tandem queuing networks and fork-join systems to analyze parallel job latency with distributed schedulers in data center cluster networks. We consider conserving policies in which a job demands system resources if the job is ready for execution. Work conserving policy states that the total backlog of work is independent of the scheduling policy at any given time. FCFS is an example of work conserving policies. Multiplexing J jobs to a single processor such that each job receives 1/J-th of the resources (or time) is another example.

The rest of the paper is organized as follows. Section 2 provides a short survey of related work in modeling and analysis of data center networks. Section 3 presents modeling and analysis of two classes of networks, Edge Sensor Networks (ESNs) and Data Center Networks (DCNs) from the queuing theory perspective. It also shows one of the contributions concerning modeling and latency analysis in data centers. It describes a queuing network model, which can be used to model the average latency and its variation. It can also be used to predict tail latency particularly at a higher load. Section 4 develops formulas for delay variation (jitter) including service time and end-to-end latency variation. It also covers factors impacting tail latency and techniques to mitigate it. Section 4.3 briefly describes trade-offs, how simulation traces were obtained and used. Finally, conclusions, limitations, and remarks are presented in Section 5.

## 2. Related Work

Recent research in the core and edge networking has focused on several aspects of underlying cluster architecture such as creating various layer 2 network topologies, designing transport protocols, virtualizing network resources, latency analysis, and so on. Both edge and core networks play a significant role in supporting enterprise networks and cloud computing services, including, but not limited to large-scale computations, web searching, email, online gaming, and social networking. As far as latency, which is a high priority in both networks, some work took an experimental approach and others tried to develop a generalized analytical model to explain and draw a trajectory of the behavior of a cluster of nodes under various workloads. For example, Flexplane [12] is an experimental platform for users to program resource management algorithms in data center networks. It is an alternative approach to simulation and a platform for experimenting in real networks. It is based on a centralized emulator on a multi-core machine. It can predict the behavior of several network schemes such as RED (Random Early Detection) and DCTCP (Data Center TCP). Unlike an analytical model, it cannot scale to support arbitrary large networks. It uses a fixed abstract packet size that may degrade accuracy with variable packet sizes.

In terms of analysis, the work in [13] surveys the research efforts conducted on applying queuing theory to model and analyze cloud computing. In [14], an approach in the context of M/G/m/m+r queue was developed to analyze a data center with heterogeneous servers. In this model, the analysis was extended to approximate the response time distributions, the mean number of tasks in the system, and the blocking probability. An interesting finding in this work is that a cloud data center that accommodates heterogeneous servers may impose longer waiting times for the incoming jobs compared to its homogeneous counterpart with the same traffic intensity. Although the results appear consistent with the prior research, the model is limited to jobs with a single task.

An approximate solution for steady-state queue length in a M/M/m system with a finite buffer has been described in [15]. A cloud platform with homogeneous servers and capacitated buffers has been modeled as a M/M/m/C queuing system to estimate the number of Virtual Machines (VMs) needed to satisfy the QoS requirements. Each server can host up to m VM instances, where *C* is the maximum queue length of a server. A load balancer, the point of entry for the system, is modeled as a M/M/1/C queue.

The authors in [16] presents an improvement to the classical model based on the power of two choices randomized load balancing. The model combines randomization techniques and static local balancing based on a round-robin policy. The model does not include queuing network delays between the dispatcher and the servers. In [17], the authors reduce the head-of-line blocking in a fat-tree network by reducing the number of exploited buffers. Neither technique consider distributed schedulers. The authors in [18] formulate an elastic aware VM (virtual machine) placement policy as an optimization problem. The model does not include network tandem queuing latency.

To improve the difficulty of building large data centers in a dense metro area, the authors in [19] proposed an optical-circuit-switched architecture that lowers cost and complexity barriers and improves scalability. In [20], the authors proposed a new solution for building a scalable and cost-effective data center networking infrastructure. The proposed topology organizes nodes in clusters of similar structure and then interconnects these clusters in a well-crafted pattern. A system of coordinates for nodes reduces the number of redundant connections between clusters while maximizing connectivity.

In terms of transport layer latency, the authors of [21] report on experiences with a congestion control technique in Google datacenters. The scheme targets an end-to-end delay by using additive increase and multiplicative decrease in window size. The tail latency for short RPCs (remote procedure calls) has been evaluated with a load close to 100%. Similarly, in [22], the performance of data center transport protocol has been investigated with a two-dimensional explicit congestion notification along with an analytical model to assess the convergence process.

## 3. Sensor and Data Center Network Models

While the M/M/1 and M/M/k described in Section 2 are helpful and simple to analyze a data center, they are not sufficient when the bottleneck is within the data center interconnection network. Hence, a network of queues is more representative to describe a data center.

**Figure 1:**
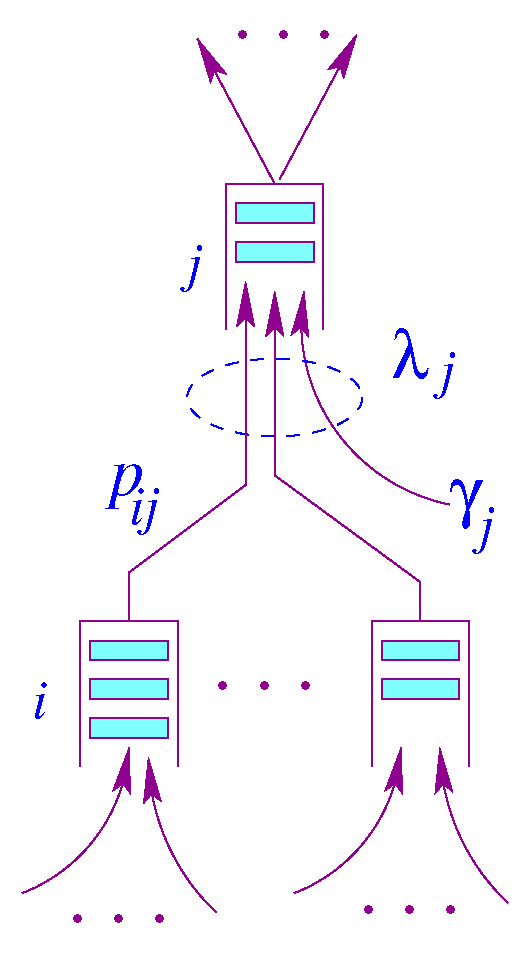
Node model for Jackson Network.

A Jackson queuing network [23] is a network of *N*  M/M/1 state-independent queuing system. Upon receiving its service at node *i*, a packet will proceed to node *j* with a probability $p_{ij}$. Figure 1 illustrates a node model for a Jackson network. The queue capacity at each node is assumed to be infinite, so there is no packet dropping.

In this model, $\gamma_{j}$ is the packet rate generated by node *j* and $\lambda_{j}$ is the aggregate rate at node *j*. Let *N* be the number of nodes in a Jackson [23] network, and $P_{N\times N}$ be a probability matrix describing routing within a Jackson network [23], where $\vec{\gamma }=\left(\gamma_{1},\gamma_{2},\cdots ,\gamma_{N}\right)$ is a vector of the exogenous mean arrival rates, and $\vec{\lambda }=\left(\lambda_{1},\lambda_{2},\cdots ,\lambda_{N}\right)$ is a vector of mean arrival rates of the traffic aggregates. Unlike the state transition used for Markov chains, the rows of P matrix need not necessarily sum up to one, i.e., $\sum_{j}p_{ij}\leq 1.$ The routing matrix P can be generated by the underlying data center interconnectivity. Assuming the network reaches equilibrium, we can write the following traffic equation using the *flow conservation principle*, in which the total sum of arrival rates entering the system is equal to the total departure rate under steady-state conditions.

(1) $$\lambda_{j}=\gamma_{j}+\sum_{i}^{N}\lambda_{i}p_{ij},\;j=1,2,\cdots ,N.$$

In the steady state,

(2) $$\vec{\lambda }=\vec{\gamma }+\vec{\lambda }P,$$

and the aggregate arrival rate vector can be solved by:

(3) $$\vec{\lambda }=\vec{\gamma }{\left(I-P\right)}^{-1}<\vec{\mu },$$

where the vector I is an identity matrix and $\vec{\mu }=\left(\mu_{1},\mu_{2},\cdots ,\mu_{{}_{N}}\right)$ is a vector representing service rates. The service times are assumed to be mutually independent and also independent of the arrival process at that queue regardless of the previous service times of the same packet in other nodes. At this point, we have the arrival rate and the service rate of each network node. From M/M/1, we can calculate the delay a packet suffers at node *j*, i.e., 1/(μ−λ), λ<μ. In the following subsections, we will compute the end-to- end delay a packet suffers by going through the Edge Sensor Network (ESN) and Data Center Network (DCN).

### 3.1. Edge Sensor Network Model

**Figure 2:**
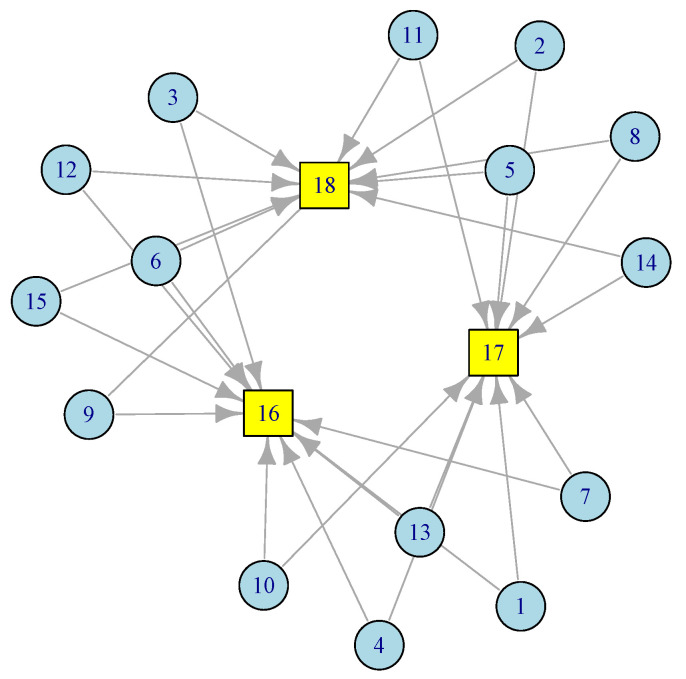
A configuration of edge sensor network with 3 gateways.

In this section, we present a two-stage tandem queuing model to characterize the Edge Sensor Network, which consists of *k* sensors or IoT nodes communicating with edge servers or gateways. We derive closed-form formulas for the key features and performance measures, including mean job response time (latency) and latency variation. Consider the sensor network in Figure 2.

In this ESN network, there are 15 sensor nodes at level 1 (ℓ=1), each has access to two gateways at level 2 (ℓ=2). Redundancy provisioning is a normal practice in Edge Sensor Networks (ESNs). The model discussed below can easily adopt any network topology. Based on the amount of traffic each node generates and the distance between sensor nodes, one can figure out the number and placement of the gateways. This is particularly useful during the deployment of sensors and smart devices in smart homes, smart roads, smart cities, smart healthcare, etc. By applying Equation (3) to the corresponding routing matrix from the network in Figure 2, we obtain

(4) $$\lambda_{s}=\left\{\begin{array}{cc} \vec{\lambda_{s}}=\left(\gamma_{{}_{1}},\gamma_{{}_{2}}\cdots \gamma_{{}_{n}}\right) & \ell =1\\ \vec{\lambda_{G}}=\vec{\lambda_{s}}\times P & \ell =2 \end{array}\right.\;T_{{}_{\ell }}=\left\{\begin{array}{cc} {\left(\mu_{h}-\gamma_{s}\right)}^{-1} & \ell =1\\ {\left(\mu_{G}-\lambda_{G}\right)}^{-1} & \ell =2 \end{array}\right.$$

To illustrate the above formulation with an example, consider that the sensor network in Figure 2 consists of 15 sensor nodes and three gateways. Further, assume $\gamma_{i}=\gamma$ and $\mu_{i}=\mu$, for an illustrative purposes, then

(5) $$\lambda_{\ell }=\left\{\begin{array}{cc} \lambda_{s}=\gamma & \ell =1\\ \lambda_{G}=5\gamma & \ell =2 \end{array}\right.\;T_{{}_{\ell }}=\left\{\begin{array}{ccc} {\left(\mu -\gamma \right)}^{-1} & \ell =1 & \gamma <\mu \\ {\left(\mu -5\gamma \right)}^{-1} & \ell =2 & \gamma <\mu /5 \end{array}\right.$$

Therefore, the end-to-end delay in the sensor network can be estimated as

(6) $$T_{ESN}=\sum_{\ell =1}^{L}T_{{}_{\ell }}=\frac{2(\mu -3\gamma )}{\left(\mu -\gamma )(\mu -5\gamma \right)}.$$

**Figure 3:**
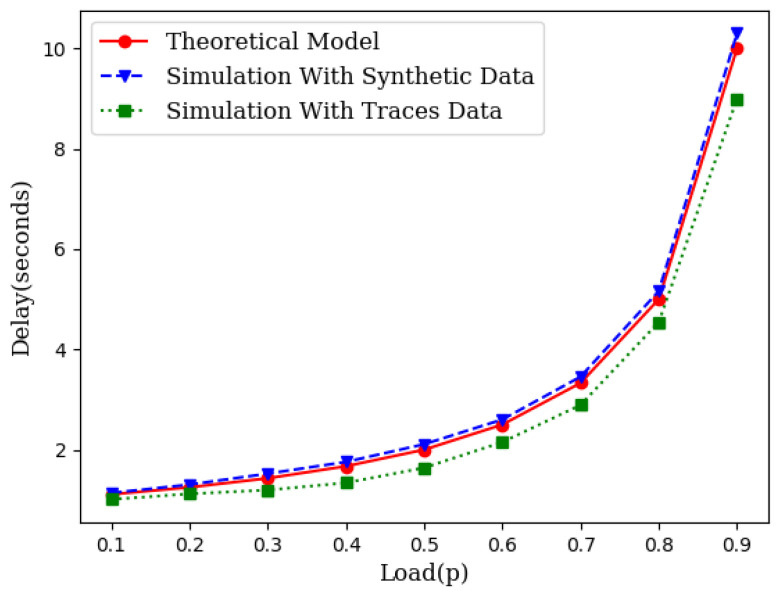
An illustration of closeness of the theoretical model with simulation.

While the theory is sound enough based on queuing theory, we have conducted two different sets of simulations. First, we generated the source traffic synthetically with an exponentially distributed inter-arrival time and an exponentially distributed service time. We varied the traffic load to compare the theoretical projection with the simulation results. We also collected traces of several IoT networks from [24]. We used the inter-arrival times and service times from these traces and conducted several runs of simulations for each load point. Figure 3 illustrates the closeness of the theoretical model with simulations which include simulations with synthetic data as well as simulations with traffic traces.

### 3.2. Simulation

To test and validate our mathematical model, we developed a simulator based on the Abstract Network Simulator (anx) [25], which is a discrete-event network simulator intended for simulations networks that exchange discrete information packets. More specifically, anx is written in pure Python3 and has only two dependencies outside Python standard libraries; NetworkX and Simpy. NetworkX is a library for creation and manipulation of complex networks, where these are modelled, viewed and manipulated as graphs. SimPy is a library for discrete-event simulations framework that relies on Python generators. anx uses NetworkX to create and manipulate network representations. These representations are converted into a computational module that SimPy can use for simulating and exchanging of information packets between nodes. The simulation model can be summarized as follows:

- The network consists of arbitrary types of nodes. A node is either a source, forwarder (edge switch), or destination node.
- Source nodes generate packets (traffic) whose destination is a randomly chosen destination node that exists in the network. Any given node generates traffic independently to other nodes.
- Each node maintains a forwarding queue where the packets are stored.
- Periodically, a node checks its forwarding queue. If there are packets in the node’s queue, it pick up a packet from the head of the queue and forwards the packet either to the destination node (if directly connected to it) or to the next-hop node along the path to the destination.
- Upon receiving a packet at the destination, the packet is processed and then removed from the network.

#### Parameters

The core simulation parameters include:

- node_pkt_rate: represents the of arrival rate (λ) of packets at nodes, except the destination.
- node_proc_delay: the time to process packet.
- node_queue_check: the time to check the forwarding queue of node. It is set to a very small value of 0.001 s.
- node_queue_cutoff: represents the length of queue of a node. It is set to 1024 packets.
- link_transm_delay: the time spent to transfer a packet between two neighboring nodes. It is set to 0.0001 s.

In terms of traffic traces, the traces were captured over different time frames of 10 min, 15 min, 30 min, 60 min, and some traces were captured over 12 h. The traces were collected using MEGA, which is a cloud-based service that can be used using all major devices and platforms from anywhere with Internet. Each packet in the trace’s files represented by starting time, source node, destination node, protocol type, and packet length.

The simulation runs for 400 s. During the simulation frame, each IoT node generated packets with exponentially distributed inter-arrival time. These packets were put into the buffer of IoT devices for transmission to the Access point (gateway). The aggregated packets at each gateway were transmitted to the Data Center Network (DCN). The simulation was executed 10 times for each load (0.1≤ρ≤0.9). The delay for each packet traversing the network was recorded and then averaged. The service time of a packet at a gateway was also exponentially distributed with the rate of μ packets per second. The transmission link delay between an IoT node and the gateway node was set to a very small value equal to 0.0001 s. The delay to monitor queue and queue length was set to 0.001 s, and each node had a queue-cutoff equal to 1024 packets.

### 3.3. Wireless Mesh Sensor Networks (WMSN) Model

**Figure 4:**
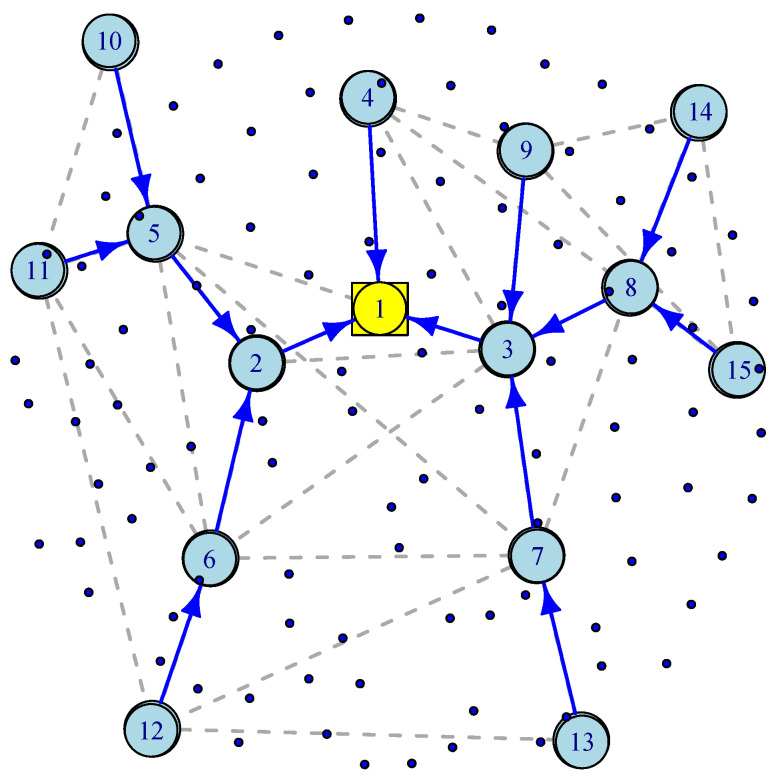
An illustration of Wireless Mesh Sensor Network.

The most challenging part of connecting IoT sensors to the infrastructure is often called “the last mile” problem that supports bridging IoT devices via a wireless mesh network to a wired Internet gateway. To better understand the WMSN’s advantages, consider wireless IP networks in which a star topology is used to communicate directly with the network through devices such as IoT gateways. If the end-to-end transmission latency is a critical metric, such as in smart highways, then a direct communication path can provide it. However, a star topology is neither fault-tolerant nor scalable. WMSNs provide an alternative solution for scalability and robustness in which alternative paths are provided through a dynamic routing mechanism such as Ad hoc On-Demand Distance Vector (AODV) Routing [26]. Figure 4 illustrates a WMSN consisting of 100 wireless sensors, 14 wireless routers, one wired gateway and a spanning tree generated by an AODV routing. In [27], the authors discuss how a gateway placement with QoS constraints can be deployed in wireless mesh networks. As in previous sections, we assume exponentially distributed inter-arrival time and service time. There are three types of nodes. Wireless sensor or IoT nodes transmit their packets to wireless routers, and wireless routers forward their packets along with the aggregated traffic received along the spanning tree towards a wireless gateway. Wireless routers act as access points for IoT nodes, sensor nodes, or mobile devices.

(7) $$\lambda_{i}=\left\{\begin{array}{ccc} \sum \gamma_{{}_{j}} & i=1, & j=1\cdots 15\\ \sum \gamma_{{}_{j}} & i=2, & j=2,5\cdots 6,10\cdots 12\\ \sum \gamma_{{}_{j}} & i=3, & j=3,7\cdots 9,13\cdots 15\\ \sum \gamma_{{}_{i}} & i=4,9\cdots 15 & \\ \sum \gamma_{{}_{j}} & i=5, & j=5,10\cdots 11\\ \sum \gamma_{{}_{j}} & i=6, & j=6,12\\ \sum \gamma_{{}_{j}} & i=7, & j=7,13\\ \sum \gamma_{{}_{j}} & i=8, & j=8,14\cdots 15 \end{array}\right.$$

(8) $$\bar{Q_{i}}=\left\{\begin{array}{cc} {\left(\mu -14\gamma \right)}^{-1} & i=1\\ {\left(\mu -6\gamma \right)}^{-1} & i=2\\ {\left(\mu -7\gamma \right)}^{-1} & i=3\\ {\left(\mu -\gamma \right)}^{-1} & i=4,9-15\\ {\left(\mu -3\gamma \right)}^{-1} & i=5,8\\ {\left(\mu -2\gamma \right)}^{-1} & i=6,7 \end{array}\right.$$

(9) $$\begin{array}{c} \bar{D}={\left(\mu -14\;\gamma \right)}^{-1}+2/5\;{\left(\mu -6\;\gamma \right)}^{-1}+\frac{7}{15}\;{\left(\mu -7\;\gamma \right)}^{-1}+\frac{8}{15}\;{\left(\mu -\gamma \right)}^{-1}\\ +2/5\;{\left(\mu -3\;\gamma \right)}^{-1}+\frac{4}{15}\;{\left(\mu -2\;\gamma \right)}^{-1} \end{array}$$

**Figure 5:**
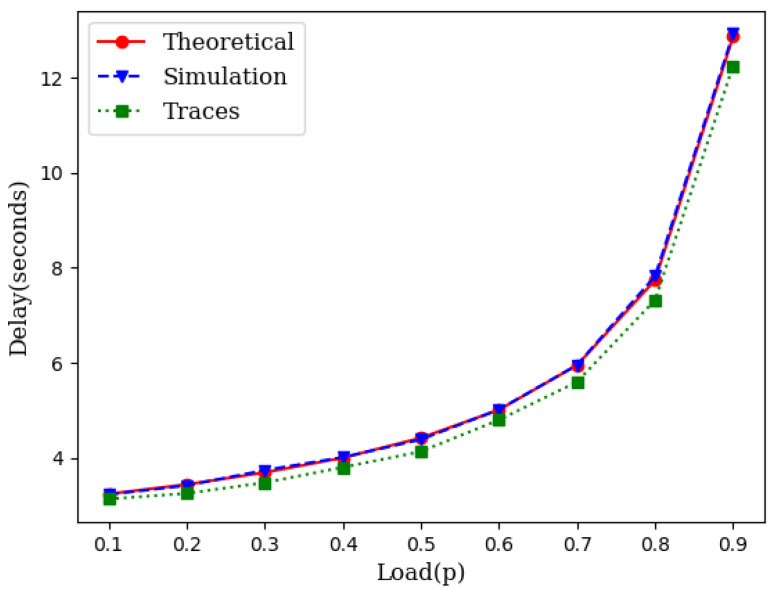
An illustration of the closeness of the theoretical model with simulation in mesh networks.

**Figure 6:**
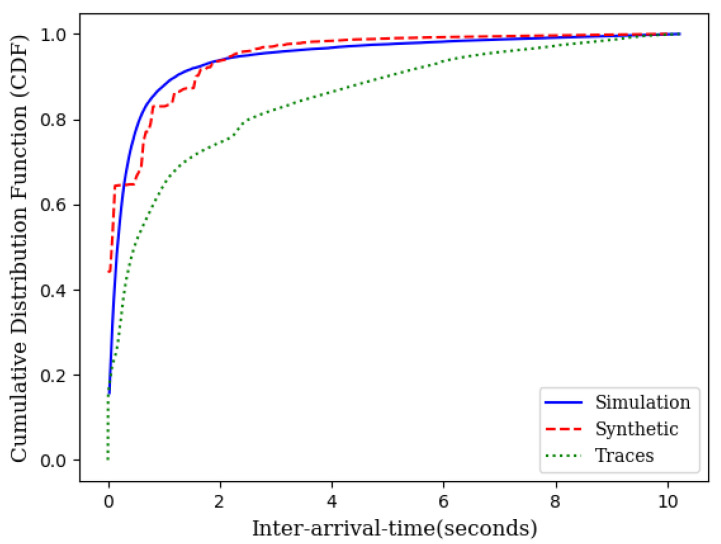
CDF of IoT traces, synthetic data, and the theoretical model.

Simulation experiments, similar to those in Section 3.1, were conducted across different loads for wireless mesh networks. Figure 5 illustrates the average delay performance with synthetic data, IoT traces and the theoretical model. The slight gap between the simulation with IoT traces and the theoretical model is the result of the gap between the CDF of the traces and the CDF of the exponential distribution as shown in Figure 6.

As it can be seen in Figure 3 and Figure 5, the theoretical delay slightly higher than the expected delay. This is due to the fact that the CDF of the inter-arrival times obtained from IoT traces is lower than the CDF of the exponential inter-arrival times with identical means but slightly different variance (Figure 6).

### 3.4. Data Center Network (DCN) Model

**Figure 7:**
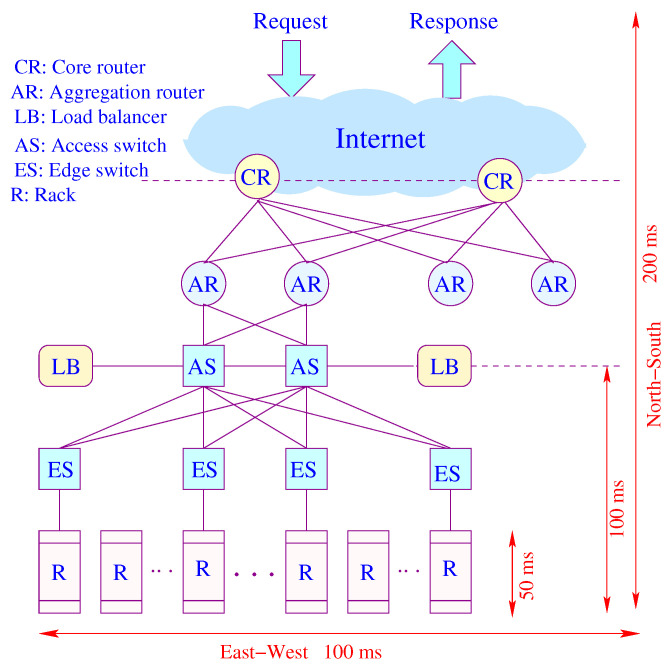
An illustration of latency components.

Figure 7 shows the conventional architecture of a data center. Requests arriving from the Internet are IP packets routed through a core router (CR) to access an aggregator router (AR), all in the layer 3 (L3) domain. A load balancer (LB) connected to a top-level access switch (AS) spreads requests evenly across multiple servers. The dominant portion of response time is consumed by the access network. Traffic moves north–south and east–west (cross-rack communications), and it is generally asymmetric in terms of latency. While spreading out reducers offers load balancing and reduces computing time, it increases communication time, congestion and tail latency. Network latency consumes data retrieval time and scalability, which is bottlenecked by latency and communication overhead. As the number of servers is increased by provisioning or scaling, the communication time increases by a higher rate than the computation time does.

From the user’s perspective, latency and its variation (jitter) are primary performance metrics. From the provider’s perspective, utilization and cost are primary performance metrics. There are a few questions this article tries to answer, including how tail latency can be mitigated without over-provisioning, how to schedule different user requests, and how these decisions help the desire to minimize energy consumption. While the answers to these questions often come from detailed experiments, it is of great value to have an analytical framework that can identify major trade-offs and challenges supporting latency-sensitive services in data center networks.

**Figure 8:**
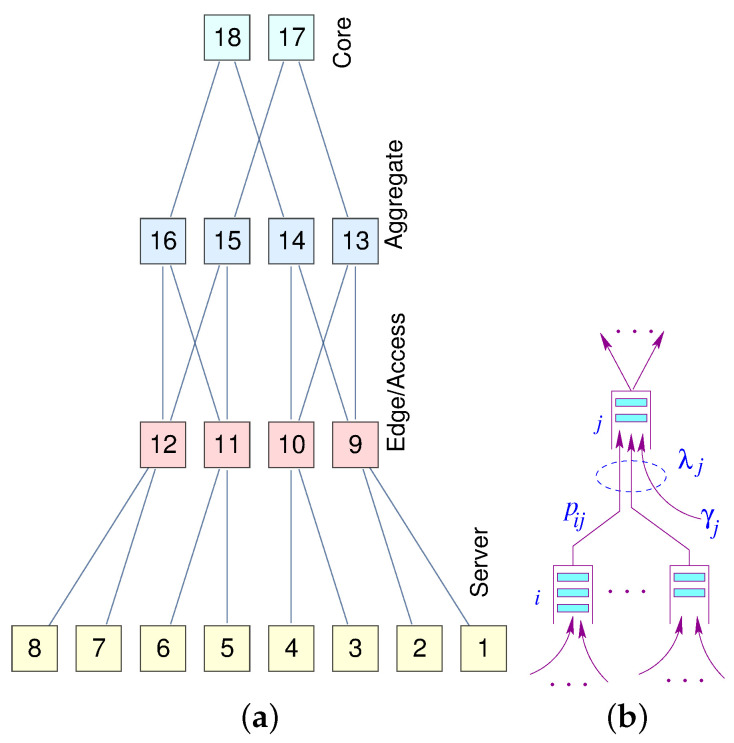
(**a**) A 4×16 fat-tree; (**b**) A node model.

Figure 8a represents a miniature fat-tree replica of a data center interconnection network. Core routers (layer 3) are interconnected with aggregate routers in a systematic block-structured form. Aggregate routers and edge switches form pods that host servers.

The delay analysis of the Data Center Network (DCN) is similar to the analysis of the Edge Sensor Network (ESN), except in the DCN there are several layers of tandem queuing systems. However, we can use the same Equation (3) to derive a closed-form solution for the mean response time. Generally, joint traffic (south–north) contributes more to the tail of latency than forked traffic (north–south). A similar approach can also be used for the traffic moving south. We extended the model developed in [6] for end-to-end delay approximation in closed-form.

Let $\lambda_{\ell j}$ be the aggregated traffic arrival at node *j* in stage *ℓ*. Given the regularity and hierarchical structure of the fat-tree, we can directly compute the load on each node at each level. Assuming a uniform distribution of traffic governed by the load balancers, we can formulate the aggregated traffic and the corresponding latency at each level (Equation (10)).

(10) $$\lambda_{\ell }=\left\{\begin{array}{cc} \vec{\lambda_{h}}=\left(\gamma_{{}_{1}},\gamma_{{}_{2}}\cdots \gamma_{{}_{n}}\right) & \ell =1\\ \vec{\lambda_{e}}=\vec{\lambda_{h}}\times P, & \ell =2\\ \vec{\lambda_{a}}=\vec{\lambda_{h}}\times P^{2}, & \ell =3\\ \vec{\lambda_{c}}=\vec{\lambda_{h}}\times P^{3}, & \ell =4 \end{array}\right.\;T_{{}_{\ell }}=\left\{\begin{array}{cc} {\left(\mu_{h}-\gamma_{h}\right)}^{-1}, & \ell =1,\;\gamma_{h}<\mu_{h},\\ {\left(\mu_{e}-\lambda_{e}\right)}^{-1}, & \ell =2,\;\lambda_{h}<\mu_{e},\\ {\left(\mu_{a}-\lambda_{a}\right)}^{-1}, & \ell =3,\;\lambda_{a}<\mu_{a},\\ {\left(\mu_{c}-\lambda_{c}\right)}^{-1}, & \ell =4,\;\lambda_{c}<\mu_{c}, \end{array}\right.$$

where *n* is the number of hosts and h,e,a,c, are indices for hosts, edge switches, aggregator, and core servers, respectively. The shortest (unique path) gives us the response time as cumulative individual delays at each level.

(11) $$T_{DCN}=\sum_{\ell =1}^{L}T_{{}_{\ell }}$$

To illustrate the above formulation with an example, consider an 8×2 fat-tree with 8 hosts, 4 edge switches, 4 aggregator switches and 2 core switches. Further, assume $\gamma_{i}=\gamma$ and $\mu_{i}=\mu$, for illustrative purposes

(12) $$\lambda_{\ell }=\left\{\begin{array}{c} \lambda_{h}=\gamma \\ \lambda_{e}=2\gamma \\ \lambda_{a}=2\gamma \\ \lambda_{c}=4\gamma \end{array}\right.\;T_{{}_{\ell }}=\left\{\begin{array}{ccc} {\left(\mu -\gamma \right)}^{-1} & \ell =1, & \gamma \leq \mu \\ {\left(\mu -2\gamma \right)}^{-1} & \ell =2, & \gamma \leq \mu /2\\ {\left(\mu -2\gamma \right)}^{-1} & \ell =3, & \gamma \leq \mu /2\\ {\left(\mu -4\gamma \right)}^{-1} & \ell =4, & \gamma \leq \mu /4 \end{array}\right.$$

(13) $$T_{DCN}=\sum_{\ell =1}^{L}T_{{}_{\ell }}=\frac{(2\mu -3\gamma )(2\mu -6\gamma )-\mu \gamma }{\left(\mu -\gamma )(\mu -2\gamma )(\mu -4\gamma \right)}.$$

The average end-to-end latency can be estimated by adding the latencies from Equations (6) and (13).

## 4. Delay Variation (Jitter) Analysis

Recent data center traffic traces [28,29] indicate that the modern applications often experience a high variability in inter-arrival time and service time. Variability in inter-arrival time represents the variability in workload, bursty or batch arrivals. Service time variability represents processing time variability due to computing, access (read/write), or communication requirements. Note that service time is the minimum task latency assuming no communication or queuing delay.

### 4.1. Service Time Variation

To investigate the impact of variability in job latency, we consider a system with a given inter-arrival time and service time distribution. We can use the squared coefficient of variation ($C^{2}$) to parameterize variability for inter-arrival time and service time.

(14) $$\begin{array}{c} C^{2}\left(t_{a}\right)=\sigma^{2}\left(t_{a}\right)/T_{a}^{2} \end{array}$$

(15) $$\begin{array}{c} C^{2}\left(t_{s}\right)=\sigma^{2}\left(t_{s}\right)/T_{s}^{2} \end{array}$$

where $t_{a}$ and $t_{s}$ denote inter-arrival time and service time, respectively. σ denotes the variance, $T_{a}$, and $T_{s}$ are the mean of inter-arrival times and service times, respectively.

The M/G/1 queuing system with exponential inter-arrival time and generic service time allows us to analyze the mean job latency $E(T_{j})$ as a function of service variability $\sigma^{2}\left(t_{s}\right)$ and mean service time $T_{s}^{2}$ via the Pollaczek–Khinchin (P–K) formula [30].

(16) $$T_{j}=T_{w}+T_{s}=\frac{\lambda (\sigma^{2}\left(t_{s}\right)+T_{s}^{2})}{2(1-\rho )}+T_{s}=\sigma^{2}\left(t_{s}\right)\cdot \frac{\lambda }{2(1-\rho )}+T_{s}\cdot \frac{2-\rho }{2(1-\rho )},$$

where $\lambda =1/T_{a}$ is the mean request arrival rate and $\rho =\lambda T_{s}$ is the normalized system load. Clearly, both $T_{s}$ and $\sigma^{2}\left(t_{s}\right)$ impact latency.

(17) $$T_{j}=\frac{C^{2}\left(t_{s}\right)}{T_{s}^{2}}\cdot \frac{\lambda }{2(1-\rho )}+T_{s}\cdot \frac{2-\rho }{2(1-\rho )}$$

(18) $$\sigma^{2}\left(t_{s}\right)=\frac{(2-\rho )(Tj-Ts)Ts}{\rho }-TjTs$$

**Figure 9:**
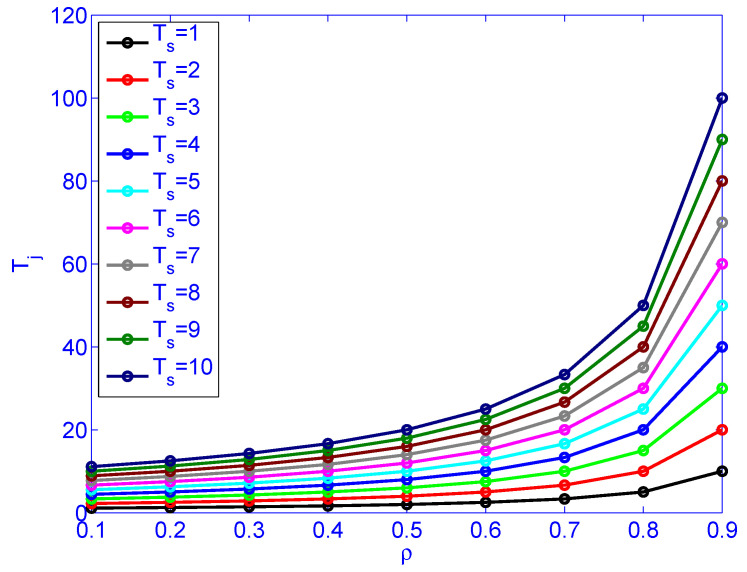
The impact of load (ρ) and service rate ($T_{s}$) on job latency $T_{j}$.

**Figure 10:**
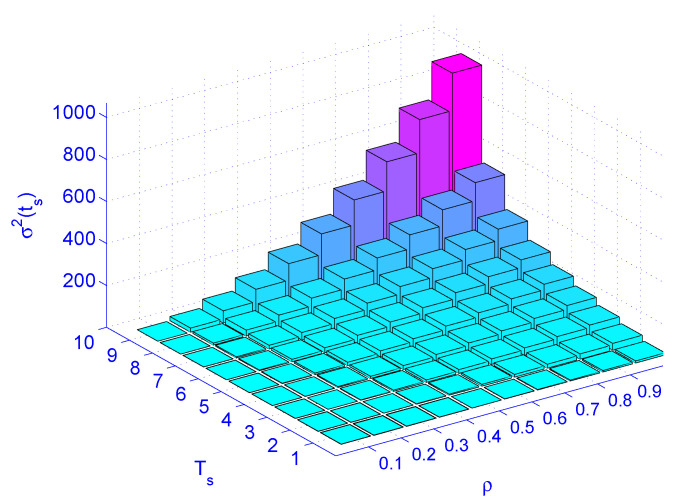
The impact of load (ρ) and service rate ($T_{s}$) on jitter $\sigma^{2}\left(t_{s}\right)$.

Figure 9 illustrates load (ρ) versus job latency for different service rates. Figure 10 illustrates load (ρ) versus service rate versus delay variation. Figure 10 indicates that the load (ρ) has more impact on the delay variation than the service time ($T_{s}$). The figure also indicates that the load has more impact on delay variation than the service rate. Distributing the load by deploying distributed schedulers gives better results than increasing the service rate (faster machines), as far as delay variation is concerned.

### 4.2. Mitigating Tail Latency

With tail latency, we are not concerned solely about the average latency. Rather, we care about the full latency distribution. At the very least, we should care about the tail. Usually, between the 90th and 99th percentiles of the tail distribution is considered. For a given fanout, how fast the children nodes have to be to get 99th percentile of latency.

Techniques such as resource provisioning, dividing jobs and parallelizing tasks, eliminating head-of-line blocking, and caching helps in reducing the tail latency. Even with a maximum level of parallelism in its finest form, the slowest instance of tasks (straggler) dominates the response time. Smaller task partitioning (micropartition) helps to reduce head-of-line blocking and achieves smoother latency distribution percentiles. At the 95th percentile latency, the scheduler sends the same request to multiple replicas (hedged requests) and using the result from the first responder. With a Canary request, one sends normal requests but falls back to hedged request if the Canary request did not finish the task in a reasonable time. More fine-grained scheduling also helps.

While parallelism mitigates tail latency, there is still a limit on how much tail latency can be improved when microservices with fork and join are used. Amdahl’s law describes the speedup (*s*) of a task when a fraction (*f*) of the computation is accelerated by a factor (*k*).

(19) $$s=\frac{1}{f/k+(1-f)}$$

**Figure 11:**
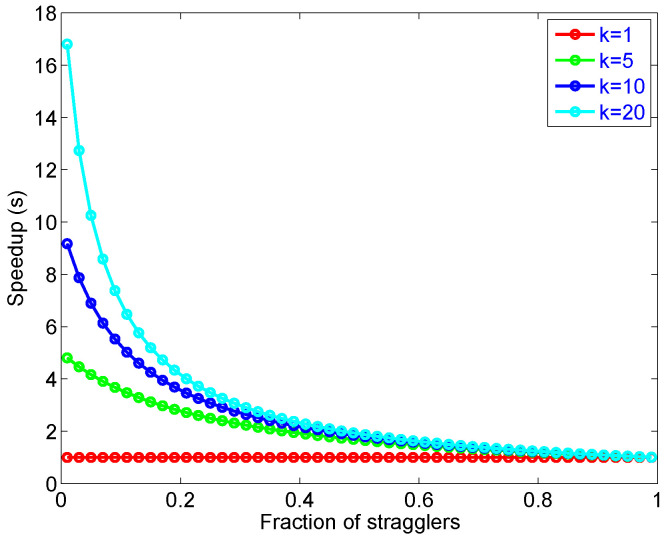
An illustration of speed-up in M/M/k limited by Amdhal’s law.

Equation (19) says that the amount of parallel speedup in a given problem is limited by the sequential portion of the problem, which are stragglers and dependent tasks. Figure 11 illustrates how stragglers can limit the speed-up gained by parallelism.

While Amdahl’s law captures the average performance from multiple cores, it does not describe tail latency. Simple models such as M/M/1 and M/M/k are particularly attractive for performance calculations in closed-form expressions. In [5], the previous analyses on Amdahl’s law for parallel and multi-core systems [31,32] have been extended to develop an analytical framework based on M/M/k queuing model to predict tail latency. The tail latency for M/M/1 can be described as the *q*-percentile of the response time. The mean response time of an M/M/1 queue is $E\left[r\right]=\frac{1}{\mu -\lambda }$, where λ is the mean arrival rate and μ is the mean service rate. Hence, the probability density and distribution functions for the latency in M/M/1 can be expressed as:

(20) $$f_{r}\left(t\right)=\left(\mu -\lambda \right)e^{-(\mu -\lambda )t},\;F_{r}\left(t\right)=1-e^{-\mu (1-\rho )t}$$

Now, the *q*-percentile of the latency can be expressed as:

(21) $$1-F_{r}\left(t\right)=\frac{q}{100}\Rightarrow t_{q}=\frac{-1}{\mu (1-\rho )}\ln\left(1-\frac{q}{100}\right)$$

For M/M/k, the probability density and distribution functions for the latency can be computed in a similar way but with more effort. However, computing the cumulative distribution function of waiting is more tractable.

(22) $$F_{r}\left(t\right)=1-P_{w}e^{-k\mu (1-\rho )t}\Rightarrow t_{q}=\frac{-\ln(\frac{100-q}{100P_{w}})}{k\mu (1-\rho )}$$

where $P_{w}$ is the Erlang-C probability of waiting.

While eliminating the sources of latency variability in large-scale systems is impractical, especially in shared environments, prediction and mitigation of tail latency are possible through queuing analysis.

### 4.3. Discussion

The experiments discussed in Section 3 and Section 4 were designed to verify the validity of the model based on the M/M/1 queuing model for Edge Sensor Networks and Data Center Networks with M/M/k servers. The average inter-arrival time, scheduling time and service time were obtained from traffic traces and fed to the Equations (6) and (13). From Google [28], Alibaba [29], and IoT traces, the CDF of various traffic parameters, such as job inter-arrival time, job size, and service time were computed directly from the traces and then used in the simulator. The DCN model gives a slightly slower response time when tested with both Google [28] and Alibaba [29] traces. An obvious reason is that the DCN model is based on an exponential inter-arrival time and an exponential service time, while the tail of the traces is longer than the tail of an exponential distribution with the same mean. It is intuitive and also observed that as network size increases (n=1250 in Google vs. n=4043 in Alibaba), the speed gap will be reduced. The traces in Google [28] and Alibaba [29] show that the average server utilization is below 40%, while we tested the DCN model across various load up to 95% to measure the impact of long-tail latency. We also observed that traces are not continuous observations. They are samples taken intermittently, and hence they are statistically suitable to be used in continuous time simulations.

## 5. Conclusions

In distributed IoT, sensors, and data center network applications, achieving predictable performance is critical, particularly in interactive jobs. Resource provisioning, such as parallel storage and multi-core servers, can pull tail latency. However, resource provisioning may be insufficient in the absence of identifying traffic characteristics and network bottlenecks. This paper, first, proposed a two-stage tandem queuing model, which is theoretically derived from a queuing-based model. This theoretical queuing network model provides a way to estimate the average end-to-end latency and predict the latency variation in closed forms. Additionally, this model introduces a feedback mechanism to investigate other major networking performance metrics, such as utilization and the optimal number of computing units needed in a single cluster. Second, a model for sensor and IoT networks was implemented, and the simulation results validate the accuracy of this model. Third, the model was extended and applied for two classes of networks, namely, Edge Sensor Networks (ESNs) and Data Center Networks (DCNs).

While parallelism reduces tail latency, there is still a limit on how much tail latency can be improved when microservices with fork and join. The takeaways from this study can be summarized as follows.

- A significant amount of tail latency can be due to queuing effects. So, reducing tandem queuing delays through distributed scheduling reduces tail latency.
- Queuing effects increase super-linearly with utilization.
- In M/M/1 and M/M/k, the tail latency can be reduced by low server utilization, i.e., over-provisioning.
- With distributed scheduling, it is possible to keep the servers busy, yet keep the tail latency low.

## Abbreviations

The following abbreviations are used in this manuscript:

DCN	Data Center Network
DCTCP	Data Center Transport Control Protocol
ESN	Edge Sensor Network
FCFS	First Come First Serve
IoT	Internet of Things
QoS	Quality of Service
REDP	Random Early Detection
TCP	Transport Control Protocol
V2I	Vehicular to Infrastructure
V2P	Vehicular to Pedestrian
V2V	Vehicular to Vehicular
